# Magnesium Nanoparticles With Pd Decoration for Hydrogen Storage

**DOI:** 10.3389/fchem.2019.00949

**Published:** 2020-02-19

**Authors:** Yana Liu, Jinglian Zhu, Zhibing Liu, Yunfeng Zhu, Jiguang Zhang, Liquan Li

**Affiliations:** ^1^College of Materials Science and Engineering, Nanjing Tech University, Nanjing, China; ^2^Jiangsu Collaborative Innovation Centre for Advanced Inorganic Function Composites, Nanjing Tech University, Nanjing, China

**Keywords:** Mg-based nanoparticles, coprecipitation, hydrogen storage, γ-MgH_2_ phase, Pd decoration

## Abstract

In this work, Magnesium nanoparticles with Pd decoration, ranging from 40 to 70 nm, were successfully coprecipitated from tetrahydrofuran (THF) solution, assigned as the Mg–Pd nanocomposite. The Mg–Pd nanocomposite exhibits superior hydrogen storage properties. For the hydrogenated Mg–Pd nanocomposite at 150°C, the onset dehydrogenation temperature is significantly reduced to 216.8°C, with a lower apparent activation energy for dehydrogenation of 93.8 kJ/mol H_2_. High-content γ-MgH_2_ formed during the hydrogenation process, along with PH_0.706_, contributes to the enhancing of desorption kinetics. The Mg–Pd nanocomposite can take up 3.0 wt% hydrogen in 2 h at a temperature as low as 50°C. During lower hydrogenation temperatures, Pd can dissociate hydrogen and create a hydrogen diffusion pathway for the Mg nanoparticles, leading to the decrease of the hydrogenation apparent activation energy (44.3 kJ/mol H_2_). In addition, the Mg–Pd alloy formed during the hydrogenation/dehydrogenation process can play an active role in the reversible metal hydride transformation, destabilizing the MgH_2_.

## Introduction

Magnesium hydride has been favorable to be applied foreground in onboard hydrogen storage systems, due to its high gravimetric hydrogen storage capacity (7.6 wt%), natural abundance, and good reversibility. Unfortunately, pure MgH_2_ shows slow sorption kinetics and high thermodynamic stability regarding the covered surface oxide layer and the low diffusion coefficient for H (Pistidda et al., 2014; Crivello et al., 2016; Yartys et al., 2019). In order to overcome these drawbacks of MgH_2_ for practical applications, numerous studies attempted to enhance the hydrogen storage performance of MgH_2_, such by as alloying Mg with other elements to alter the thermodynamic stability, doping with additives or catalysts, and reducing Mg particles to nano scale. “Nanosize effect” has been considered as an approach with the potential of leading to improvements in both kinetics and thermodynamics (Schneemann et al., 2018), which is based on (i) a larger surface area and thus more hydrogen dissociation sites (ii) the shortened hydrogen diffusion distances which in turn enhance kinetics, and (iii) the increased number of atoms at grain boundaries to enhance the hydrogen diffusion rates (Yao et al., 2011; Schneemann et al., 2018; Sun et al., 2018). Recently, an adapted Rieke method has emerged as an alternative to synthesize Mg nanoparticles to dramatically enhance the hydrogen sorption kinetics of Mg. For instance, Jeon et al. (2011) reported the synthesis of air-stable Mg nanoparticles (~4.9 nm) embedded in PMMA matrix, which enable both the storage of a high hydrogen capacity and rapid kinetics. Norberg et al. (2011) prepared Mg nanoparticles with controllable sizes by a similar method, and the ab-/desorption hydrogen kinetics were proven to be dramatically faster for nanoparticles with smaller sizes, as a result of the increase of the defect density formed in smaller nanoparticles. Recently, we have investigated that the Mg–TM (TM = Ni, Ti, Fe, Co, and V) nanocomposites can be coprecipitated from solution, in which the coprecipitated Ni, Ti, Fe, V, or Co has high catalytic efficiency in improvement of the ab-/desorption hydrogen kinetics of Mg nanoparticles (Liu et al., 2014a,b, 2015). The adapted Rieke method is considered as a better method for the preparation of nano-sized Mg with controllable sizes, and which can dope transition metal catalysts into a Mg/MgH_2_ system by a simple chemical route. With regard to the nanoscaling, various catalysts for improving the hydrogen storage properties of MgH_2_ have been investigated, such as transition metals/metal oxides/halogenides (Ma et al., 2009, 2013; Cui et al., 2014; Rizo-Acosta et al., 2018; Zhang et al., 2018; Liu et al., 2019b), rare earth metal oxides/halogenides (Singh et al., 2013; Lin et al., 2014, 2015; Wang et al., 2015), carbon materials (Lukashev et al., 2006; Liu et al., 2013; Rather et al., 2016), and other hydrides (Lu et al., 2010; Terent'ev et al., 2015; Jangir et al., 2018). In addition, some noble metals, such as Pd, have been reported to be effective for facilitating hydrogenation of MgH_2_ (Du et al., 2007; Callini et al., 2010; Ham et al., 2013; Chung et al., 2015; Liu et al., 2019a). Liu et al. (2019a) reported that elemental Pd played a dominant role in accelerating the preferential diffusion of hydrogen atoms at the Pd/Mg interface, during the hydrogenation process. Callini et al. (2009) suggested the occurrence of Mg-alloying as a path for subsequent feasible H-exchange, when Pd was deposited on top of a thick MgO layer surrounding the Mg NPs core. Du et al. (2007) reported that the Pd-dopant provides a much lower activation barrier for both dissociation of molecular hydrogen and diffusion of atomic hydrogen on the Mg surface, based on ab initio density functional theory calculations.

Inspired by the above considerations, a Mg–Pd nanocomposite was coprecipitated from THF solution. The Mg nanoparticles were homogeneously decorated with nano-sized Pd. The hydrogen sorption properties were investigated, and the catalytic mechanisms of Pd were also proposed.

## Materials and Methods

### Sample Preparation

As early as in 1972, Rieke et al. reported a general procedure for the preparation of highly reactive magnesium metal, which was prepared by reducing a magnesium halide with alkali metal in an ethereal solvent (Rieke and Hudnall, 1972). In this work, anhydrous MgCl_2_, PdCl_2_, lithium naphthalide (LiNp), and tetrahydrofuran (THF) solution were introduced to prepare the Mg–Pd nanocomposite by the coprecipitation method (an adapted Rieke method). During the sample preparation process, the real reducing agent is metal Li, while naphthalene is used as an electron carrier. Due to the presence of naphthalene, metal Li would dissolve in THF, forming a blackish green solution, which can speed up the reduction reaction of active Mg. In raw materials, Pd:Mg was kept in a weight ratio of 1:9. The reaction processes are as follows:

(1) $$\begin{array}{r} {\text{MgCl}}_{2}+2\text{Li}\to {\text{Mg}}^{*}+2\text{LiCl} \end{array}$$

(2) $$\begin{array}{r} {\text{PdCl}}_{2}+2\text{Li}\to {\text{Pd}}^{*}+2\text{LiCl} \end{array}$$

Naphthalene (5.52 g) and lithium (0.299 g) were dissolved in the freshly distilled THF (50 ml), forming a homogeneous blackish green solution (LiNp/THF). A mixed THF solution of MgCl_2_ and PdCl_2_ was prepared by anhydrous MgCl_2_ (1.904 g) and PdCl_2_ (0.09 g) dissolved in 200 ml THF at 60–70°C. The coprecipitation reaction process and the separation and drying process of nanocomposites were performed with reference to a relevant literature report (Liu et al., 2014b).

### Sample Characterization

The crystal structure and the phase composition of the samples were analyzed by X-ray diffraction (XRD) with Cu Kα radiation (40 kV and 35 mA) using an ARL X'TRA diffractometer. The morphology and microstructure of as-prepared samples were observed by using a JEM-2100F transmission electron microscope (TEM). A Sieverts-type apparatus (AMC) was used to examine the hydrogen storage properties. The auto pressure—composition—temperature (PCT) measurements were performed under a hydrogen pressure up to 4 MPa. The hydrogen absorption kinetics was characterized under an initial hydrogen pressure of 3.0 MPa. The dehydrogenation experiments were performed using diffraction scanning calorimetry (DSC, Netzsch STA 449F3) at various heating rates under Ar gas flow.

## Results and Discussion

### Microstructural Characterization

**Figure 1:**
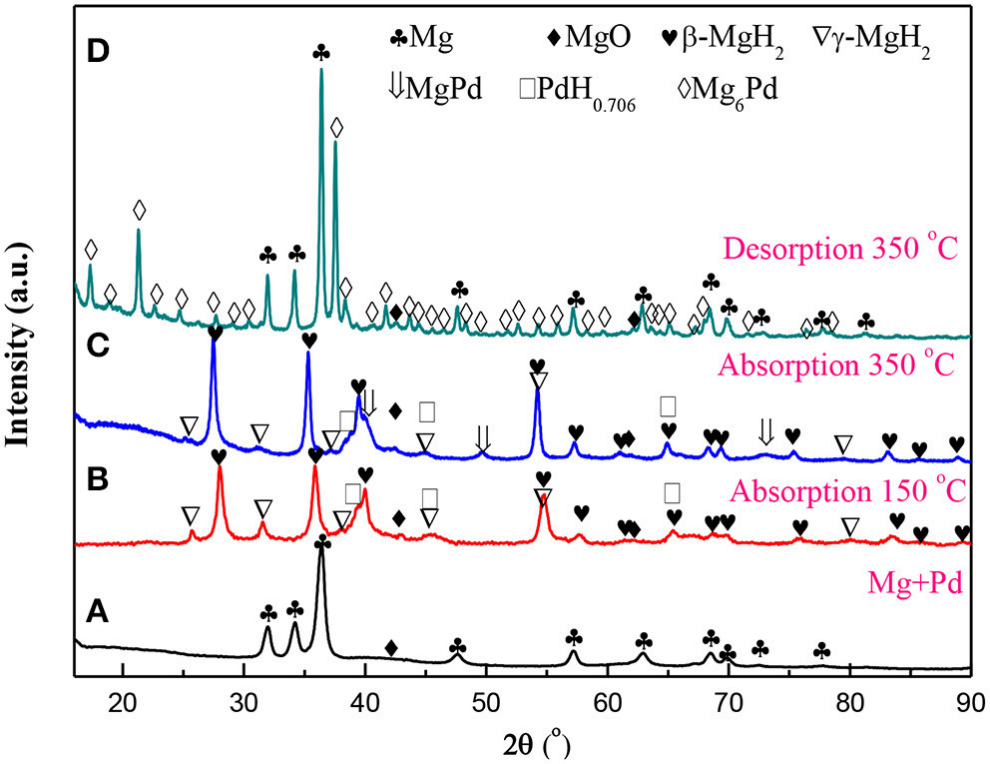
X-ray diffraction (XRD) patterns for the Mg–Pd nanocomposite in varying states. **(A)** As prepared, **(B,C)** hydrogenated at 150 and 350°C for 2 h, and **(D)** dehydrogenated at 350°C for 2 h.

Figure 1 presents the XRD patterns of the Mg–Pd nanocomposite in varying states. As shown in Figure 1A, almost all diffraction peaks of as-prepared nanocomposite can be characterized with the hexagonal Mg phase. Only a weak peak at ~42.9° can be indexed with MgO, and the characteristic diffraction peaks of Pd are absent. A small amount of MgO is likely due to the oxidation in the process of sample preparation for the XRD analysis, as a result of high chemical activity of Rieked Mg. The absence of Pd peaks suggests that Pd synthetized from solution is in the form of extremely fine particles or amorphous, and hence, it does not generate detectable diffraction intensity. A similar phenomenon has been found in our previous research work (Liu et al., 2014a, 2015). The lattice parameters of the Mg phase can be refined to be a = b = 0.3225 nm, and c = 0.5242 nm for the Mg–Pd nanocomposite, which are slightly larger than those of pure Mg reduced by the same method (a = b = 0.3196 nm, c = 0.5199 nm; Liu et al., 2014b), indicating that a part of the Pd atoms may get into the crystal lattice of the Mg during the preparation, resulting in the lattice expansion of Mg. In addition, the average crystallite of Mg can be determined to be about 22.5 nm by the Scherrer equation. When the Mg–Pd nanocomposite was hydrogenated under 3 MPa hydrogen pressure at 150°C for 2 h, the majority phases of the hydrogenated Mg–Pd nanocomposite are the tetragonal β-MgH_2_ and the orthogonal γ-MgH_2_, along with a small amount of MgO and PdH_0.706_ phases, as shown in Figure 1B. The metastable γ-MgH_2_, belonging to a-PbO_2_-type orthorhombic crystal structure, is usually produced by ball milling (Varin et al., 2006; Ponthieu et al., 2013; Zhou et al., 2015) as well as under high compressive stress (Vajeeston et al., 2006; Siviero et al., 2009; Ham et al., 2013). Zhou et al. (2013) pointed out that the H octahedrons surrounding Mg atoms are deformed in the γ-MgH_2_. Besides, the octahedral chain takes a zigzag form, while for the β-MgH_2_, the chain is straight. Sander et al. studied hydrogen transport kinetics in β- (termed α-MgH_2_ in their study) and γ-MgH_2_ based on theoretical calculations and found that H vacancies' concentrations are higher and more mobile in the γ-MgH_2_ than in the β-MgH_2_ (Sander et al., 2016). Therefore, we can speculate that the metastable γ-MgH_2_ structure is likely due to the deformations and structural defects formed in the MgH_2_ lattice during the preparation (ball milling or under high compressive stress). The formation of the γ-MgH_2_ phase is also found in the Mg–TM (TM = Ni, Ti, Fe, Co, and V) nanocomposites coprecipitated from solution (Liu et al., 2014a,b, 2015). Norberg et al. (2011) proposed that a high density of defects may form in the Mg lattice through the adapted Rieked method. In addition, Xiao et al. (2016) synthesized β-/γ-MgH_2_ nanocomposites via a simple wet chemical route by ball milling MgH_2_ with LiCl as an additive at room temperature followed by THF treatment and revealed that THF solution plays a vital role in the synthesis of the γ-MgH_2_ phase. In this work, high-density defects formed in the nanocomposite and THF solution effect may contribute to the formation of the γ-MgH_2_ phase at lower temperature. On hydrogen loading, the PdH_0.706_ peaked at 38.7°C, corresponding to the (111) plane. The PdH_0.706_ phase also could be found when Au/Mg/Pd films were hydrogenated at 200°C (Akyildiz et al., 2006). As the temperature was increased to 350°C, in addition to the main phase of β-MgH_2_, MgPd, PdH0.706, and γ-MgH_2_ phases were clearly detected in Figure 1C. The appearance of the Pd-richer intermetallic compound MgPd indicates that Pd is inter-mixing with Mg at the Pd–Mg interface, which is considered to be the equilibrium end product (Callini et al., 2010). The amount of γ-MgH_2_ phase in hydrogenated nanocomposite decreased with increasing the hydrogenation temperature, due to the removal of the lattice deformation and structural defects in Mg at high temperature. Shena and Aguey-Zinsou, reported that the γ-MgH_2_ was fully converted to β-MgH_2_ after hydrogen cycling at 200°C, due to the annealing of defects and relief of strain (Shena and Aguey-Zinsou, 2017). After dehydrogenation at 350°C for 2 h under 0.05 MPa hydrogen pressure, the phases of Mg, MgO, and Mg_6_Pd could be detected in the XRD pattern of Figure 1D. The formation of Mg_6_Pd confirms an interaction between MgH_2_ and MgPd or PdH_0.706_ during the process of dehydrogenation, as in following equations:

(3) $$\begin{array}{r} \text{MgPd}+5{\text{MgH}}_{2}\to {\text{Mg}}_{6}\text{Pd}+5{\text{H}}_{2} \end{array}$$

(4) $$\begin{array}{r} {\text{PdH}}_{0.706}+6{\text{MgH}}_{2}\to {\text{Mg}}_{6}\text{Pd}+7.412{\text{H}}_{2} \end{array}$$

Huot et al. (2009) found that the bulk Mg_6_Pd could reversibly absorb hydrogen in three disproportionation reactions and finally be transformed to the MgPd phase again. MgPd or Pd_H0.706_ interacting with MgH_2_ may alter the thermodynamics of the dehydrogenation of MgH_2_. Therefore, the thermodynamics of the Mg–Pd nanocomposite will be discussed in greater detail later.

**Figure 2:**
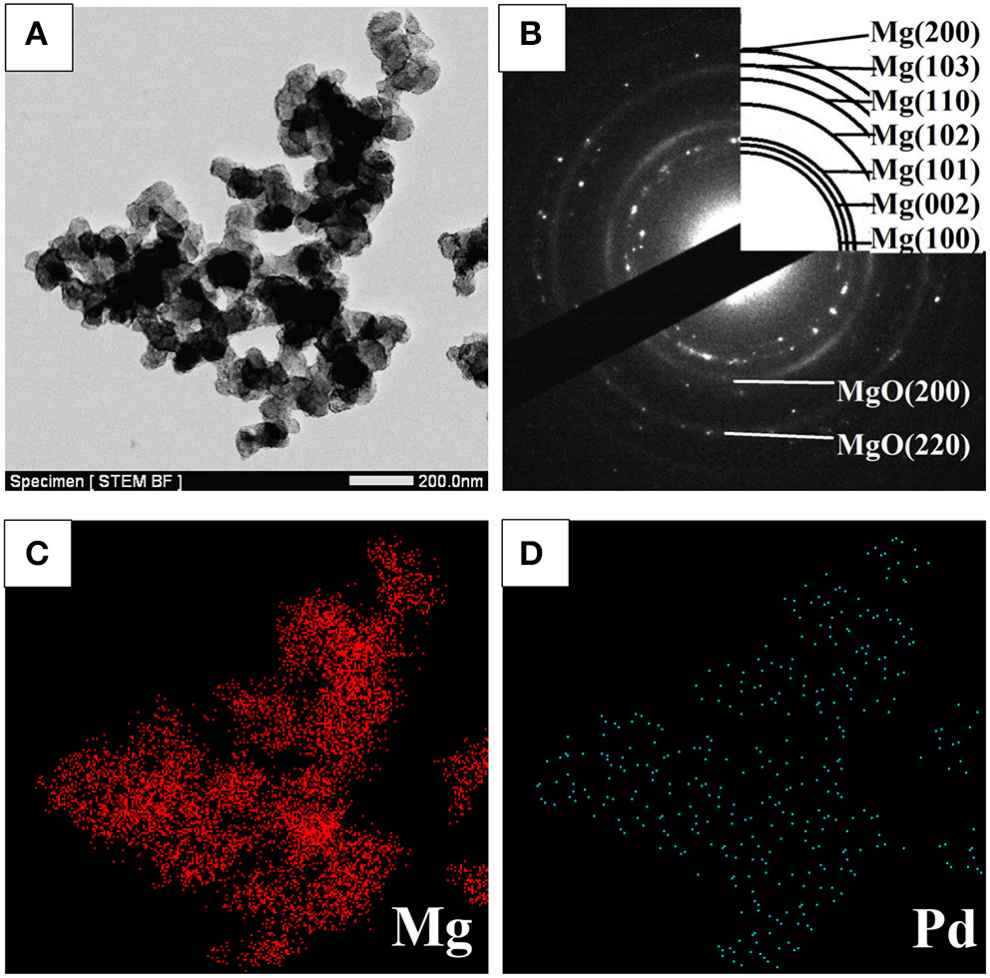
**(A)** STEM micrograph and **(B)** SAED pattern of the Mg–Pd nanocomposite, along with **(C,D)** EDS elemental maps of Mg and Pd.

**Figure 3:**
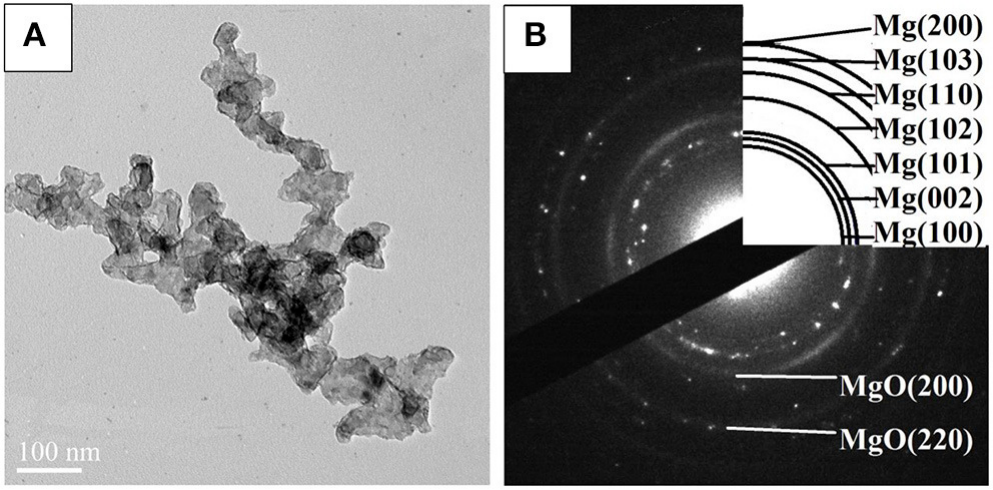
**(A)** Transmission electron microscope (TEM) micrograph and **(B)** SAED pattern of the Mg–Pd nanocomposite hydrogenated at 150°C for 10 h.

STEM micrographs and SAED pattern of the Mg–Pd nanocomposite are shown in Figure 2. The Mg–Pd nanocomposite is composed of irregularly shaped particles aggregating together, with their particle sizes ranging from 40 to 70 nm, as shown in Figure 2A, while for the pure Mg without doping catalysts, the nanoparticles were plate-shaped and stacked together forming a shape like a “chicken claws” structure (Liu et al., 2014b). The above result indicates that doping with Pd may dramatically affect the preferred orientation of Mg particle growth. As shown in the corresponding SAED pattern (Figure 2B), the diffraction rings or points can be indexed with Mg and MgO phase, without Pd, which is in accordance with the XRD result. In order to qualitatively evaluate the distribution of Pd in Mg particles, EDS elemental maps of Mg and Pd were performed. As can be seen, Pd is homogeneously distributed on the surface or inside Mg particles, without obvious reunion phenomenon. The actual Pd weight content in the nanocomposite is about 5 wt%, much lower than the initial doping amount. Only half the amount of Pd was embedded in or attached on the surface of Mg particles, and the rest might be lost during the centrifugation and repeated washing processes. The TEM micrograph and the corresponding SAED pattern of the Mg–Pd nanocomposite hydrogenated at 150°C for 2 h are shown in Figures 3A,B, respectively. As can be seen, the particle size of the hydrogenated Mg–Pd nanocomposite ranges from 20 to 30 nm, much lower than that hydrogenated before, which is likely due to a large number of nucleation sites at the Mg–Pd interface and high density of defects on the Mg particles. The same phenomenon was found in the previously reported Mg–TM (TM = Ni, Ti, Fe, Co, and V) nanocomposites (Liu et al., 2014a,b, 2015). As shown in the STEM images, Pd is homogeneously distributed on the surface or inside Mg particles, without obvious reunion phenomenon. Kumar et al. has reported that multinucleation sites for the Mg → MgH_2_ hydrogenation process could be created by the uniformly distributed catalyst on the Mg nanoparticle surface, which could prevent grain growth (Kumar et al., 2017). A high density of defects on the Mg particles could also be found in the previously reported Mg–TM (TM = Ni, Ti, Fe, Co, and V) nanocomposites (Liu et al., 2014a,b, 2015) and Mg nanocrystals prepared by Norberg et al. (2011), which could provide more nucleation sites for the Mg → MgH_2_ hydrogenation process. In addition, the particles' growth trend is not obvious at lower hydrogenation temperature (150°C), resulting in the refinement of MgH_2_ particles. As shown in Figure 3B, no diffraction rings or points can be indexed with the β-/γ-MgH_2_ phase, suggesting that the electron beam induces full dehydrogenation of MgH_2_ during the TEM measurement under high-vacuum conditions. Besides, Pd or PdH_0.706_ is not found, maybe due to the poor crystallinity.

### The Effects of Mg_6_Pd on the Thermodynamics

**Figure 4:**
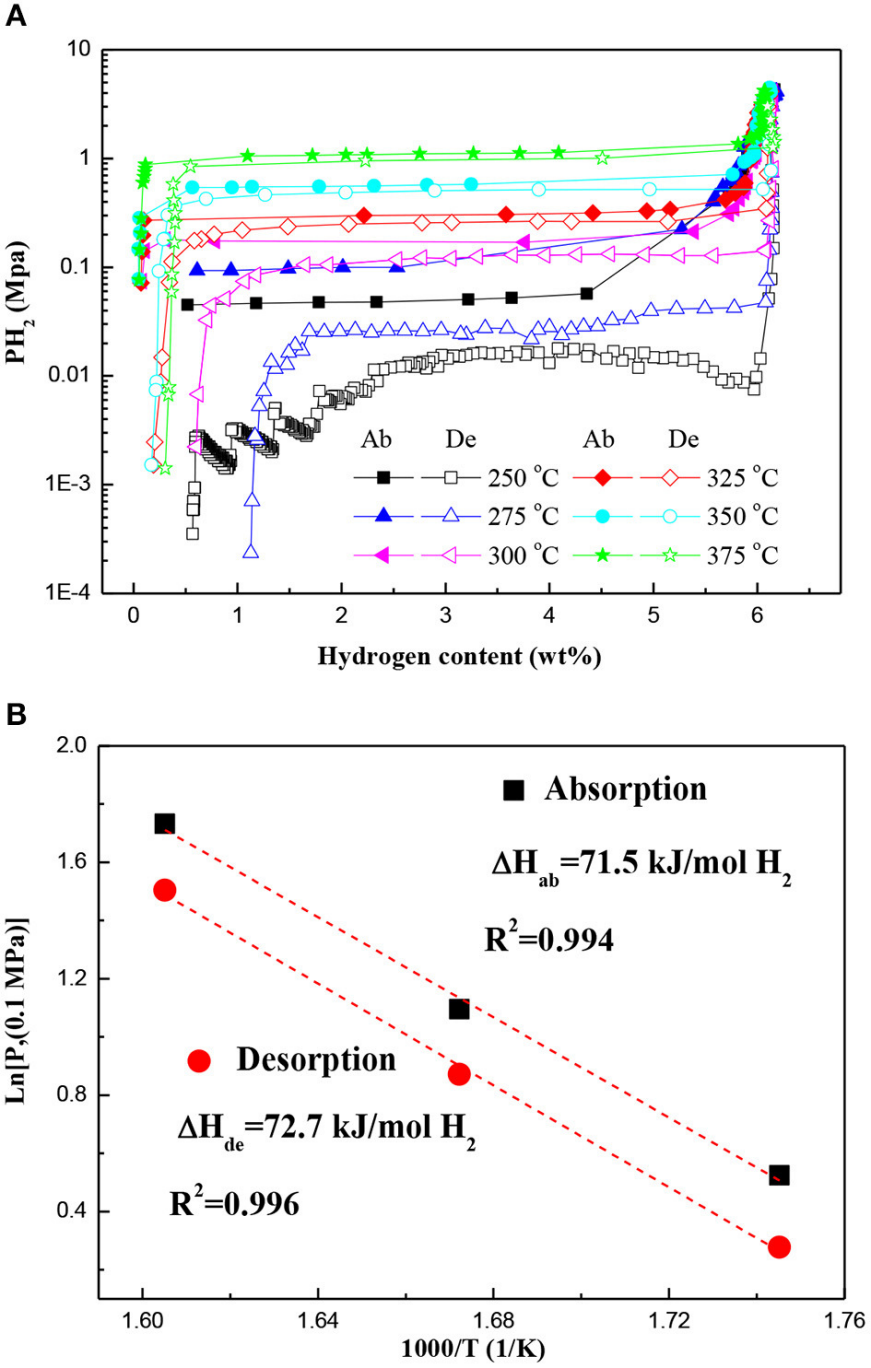
**(A)** PCT curves of the Mg–Pd nanocomposite measured at different temperatures and **(B)** the corresponding van't Hoff plots. Ab, absorption; De, desorption.

Figure 4A displays the PCT curves of the Mg–Pd nanocomposite measured at different temperatures in a hydrogen pressure range from 0.02 to 4 MPa. The first hydrogen cycle at 350°C was conducted for the test sample to obtain a Mg–Mg_6_Pd system. It can be found that only one plateau is present across the whole range of the hydrogen content in each PCT curve. Obvious plateaus of Mg_6_Pd cannot be clearly identified. However, previous investigation (Huot et al., 2009) showed that the hydrogen absorption process of Mg_6_Pd was divided into three stages:

(5) $$\begin{array}{r} {\text{Mg}}_{6}\text{Pd }+2.35{\text{H}}_{2}↔2.35{\text{MgH}}_{2}+{\text{Mg}}_{3.65}\text{Pd} \end{array}$$

(6) $$\begin{array}{rc} 2.35{\text{MgH}}_{2}+{\text{Mg}}_{3.65}\text{Pd}+1.15{\text{H}}_{2}↔3.5{\text{MgH}}_{2}\\ + & \;0.5{\text{Mg}}_{2}{\text{Pd}}_{5} \end{array}$$

(7) $$\begin{array}{r} 3.5{\text{MgH}}_{2}+0.5{\text{Mg}}_{2}{\text{Pd}}_{5}+1.15{\text{H}}_{2}↔\text{MgPd}+5{\text{MgH}}_{2} \end{array}$$

A higher plateau should appear in the PCT curve, which is attributed to the transformation Mg_6_Pd + 5H_2_ ↔ MgPd + 5MgH_2_. In addition, Shena and Aguey-Zinsou (2017) reported that P(H_2_)_abs_ = 5 MPa is not sufficient to induce the formation of MgPd at the higher temperature of 350°C. In fact, at 350°C, 3 MPa is enough for the formation of MgPd, as shown in Figure 1C. This is in accordance with the one plateau observed in the PCT curve for the Mg–Mg_6_Pd system, suggesting an excellent synergistic effect for Mg and Mg_6_Pd during hydrogenation/dehydrogenation processes. The corresponding van't Hoff plot (ln P vs. 1/T) is used to estimate hydrogenation and dehydrogenation enthalpies, as shown in Figure 4B. The hydrogenation and dehydrogenation enthalpies are determined to be −71.5 and 72.7 kJ mol^−1^ H_2_, respectively, which are slightly lower than that of pristine MgH_2_ (74.7 kJ mol^–1^ H_2_; Crivello et al., 2016). Zhang et al. found that MgH_2_ is preferentially decomposed by virtue of the Mg–Pd alloy instead of self-decomposition, which can reduce thermal stability of the MgH_2_ through interacting with MgH_2_ and forming the Mg_6_Pd phase (Zhang et al., 2015). Therefore, it is reasonable to suggest that the Mg–Pd alloy plays an active role in the reversible metal hydride transformation, destabilizing the MgH_2_.

### The Effects of γ-MgH_2_ and PdH_0.706_ on Dehydrogenation Performances

**Figure 5:**
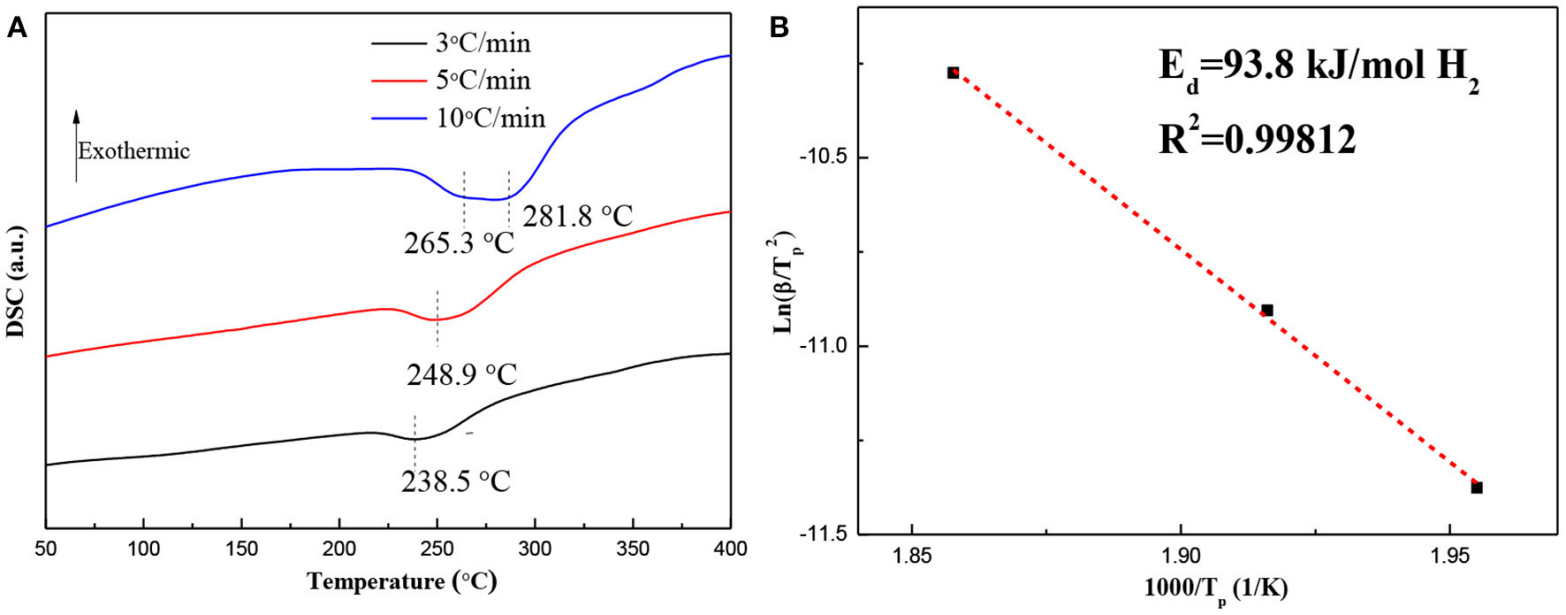
**(A)** Diffraction scanning calorimetry (DSC) curves and **(B)** the corresponding ln(β/T_*p*_^2^) −1,000/T_p_ plots for the hydrogenated Mg–Pd nanocomposite at different heating rates.

DSC curves and the corresponding ln(β/*T*_*p*_^2^)−1,000/*T*_*p*_ plots for the hydrogenated Mg–Pd nanocomposite are shown in Figure 5. The Mg–Pd nanocomposite was hydrogenated at 150°C for 2 h, as the test sample. Figure 5A shows that there is a broader and asymmetrical endothermic peak corresponding to desorption of the γ-MgH_2_ and β-MgH_2_ phases in 238.5 and 248.9°C at heating rates of 3 and 5°C/min, respectively, while for the heating rates of 10°C/min, the final endothermic peak is a superposition of two contributing peaks of 265.3 and 281.8°C. The above results suggest that γ-MgH_2_ decomposes along with the β-MgH_2_, which may trigger the decomposition of the β-MgH_2_ during the dehydrogenation process. The onset dehydrogenation temperature at a heating rate of 3°C/min is reduced to 216.8°C, representing a 94.3°C reduction as compared with the pure MgH_2_ prepared through the same method (Liu et al., 2014b), and for the reported Mg–TM (TM = Ni, Ti, Fe, Co, and V) nanocomposites, the onset dehydrogenation temperatures were determined to be 244.6, 296.1, 246.5, 249.1, and 278.2°C, respectively (Liu et al., 2014a,b, 2015). The hydrogenated Mg–Pd nanocomposite exhibits superior hydrogen desorption kinetics compared with the previously reported Mg–TM (TM = Ni, Ti, Fe, Co, and V) nanocomposites. The pure MgH_2_ is a single phase of β-MgH_2_, without doping catalyst. The γ-MgH_2_ phase is known to be less stable than the β-MgH_2_ phase. The plane (110) of γ-MgH_2_ has a predicted enthalpy of 44.66 kJ mol^−1^ H_2_, while for the same β-MgH_2_ plane, the enthalpy is 78.16 kJ mol^−1^ H_2_ (Zhou et al., 2015). In addition, the heavily distorted MgH_6_ octahedron in the γ-MgH_2_ potentially can facilitate hydrogen diffusion (Sander et al., 2016). Shena and Aguey-Zinsou (2017) proposed that γ-MgH_2_ can further facilitate hydrogen desorption from β-MgH_2_, which can act as a pathway for faster hydrogen diffusion. In this work, a higher content of γ-MgH_2_ is achieved in the hydrogenated products, as shown in Figure 1B. Therefore, it can be concluded that the γ-MgH_2_ phase can significantly reduce the dehydrogenation temperature and improve the dehydrogenation kinetics by acting as a pathway for faster hydrogen diffusion. The PdH_0.706_ phase in the hydrogenated products also is reported to be effective for improving desorption hydrogen kinetics and reducing the hydrogen desorption temperature, which may help the recombination process of H atoms into hydrogen molecules on the surface (Tang et al., 2008). Higuchi et al. (2002) reported that “cooperative phenomena” can explain the significant improvement in the dehydriding properties of the Pd/Mg/Pd films, in which hydrogen exhibits elastic interactions between MgH_2_ and PdH_0.6_. The activation energy, *E*_*d*_, for the hydrogen desorption mechanism of the hydrogenated Mg–Pd nanocomposites is estimated by the Kissinger equation:

(8) $$\begin{array}{r} \text{ln}\left(\beta /T_{p}^{2}\right)=\text{A}-E_{d}/\left(\text{R}T_{p}\right) \end{array}$$

where β is the heating rate, *T*_*p*_ is the peak temperature, and A is a linear constant.

Figure 5B shows the plot of ln(β/$T_{p}^{2}$) vs. 1,000/*T*_*p*_ for the hydrogenated Mg–Pd nanocomposites. The *E*_*d*_ value can be determined to be 93.8 kJ/mol H_2_, which is much lower than that of the hydrogenated pure Mg (147.4 kJ/mol H_2_; Liu et al., 2014b) and the reported Mg–TM (TM = Ni, Ti, Fe, Co, and V) nanocomposites (139.1, 170.9, 118.1, 110.1, and 147.7 kJ/mol H_2_, respectively; Liu et al., 2014a,b, 2015). The lower dehydrogenation temperature and *E*_*d*_ value indicate that γ-MgH_2_ and PH_0.706_ phases formed during the hydrogenation process contribute to the enhancing desorption kinetics.

### The Effects of Pd on Lower Temperature Hydrogenation Performances

**Figure 6:**
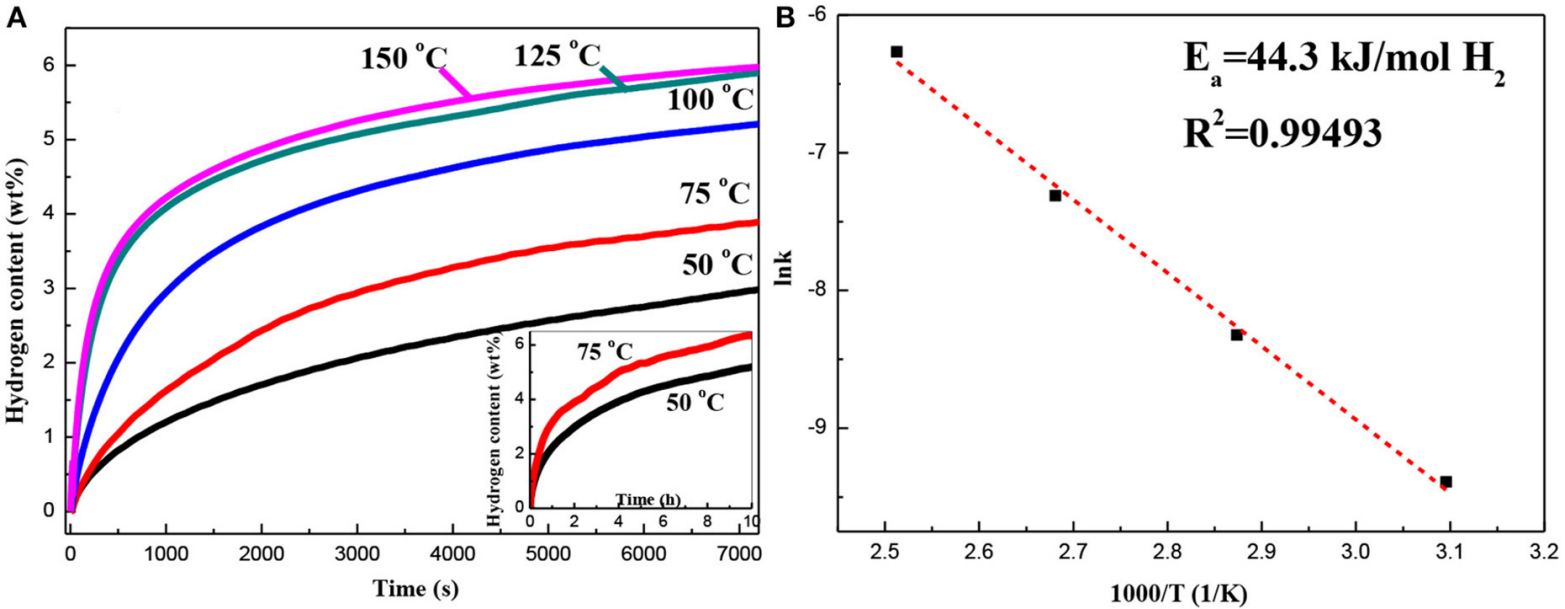
**(A)** Hydrogen absorption curves of the Mg–Pd nanocomposite measured at different temperatures for 2 h and **(B)** the corresponding ln*k* −1,000/*T* plot.

Figure 6A shows the first isothermal hydrogen absorption curves of the Mg–Pd nanocomposite measured at 50, 75, 100, 125, and 150°C. As can be seen, the Mg–Pd nanocomposite exhibits excellent hydrogen absorption performance. The hydrogen storage capacity can reach up to 3.0 wt% in 2 h at 50°C. In contrast, the hydrogen capacity of the pure Mg is 2.6 wt% within 2 h at a higher temperature of 125°C. The result suggests that Pd exhibits high catalytic activity on accelerating the hydrogenation rate of Mg. The apparent activation energy of dehydrogenation can be calculated based on the Johnson–Mehl–Avrami–Kolmogorov (JMAK) model and the Arrhenius theory (Liu et al., 2014a,b, 2015). The Arrhenius-type plot of ln*k* vs. 1,000/*T* is drawn in Figure 6B. Thus, *E*_*a*_ of the Mg–Pd nanocomposite can be determined to be 44.3 kJ/mol H_2_, much lower than that of the pure Mg (73.1 kJ/mol H_2_; Liu et al., 2014b). Such a low energy barrier explains the excellent absorption kinetics of the Mg–Pd nanocomposite at low temperatures. As shown in the STEM images (Figure 2), Pd is homogeneously distributed on the surface or inside Mg particles, with the particle size ranging from 40 to 70 nm. The reported theoretical calculations indicate that the Pd-dopant can provide a much lower activation barrier for both dissociation of molecular hydrogen and diffusion of atomic hydrogen on the Mg surface (Du et al., 2007). Pd in the form of small particles dispersed on the Mg particles can catalyze the dissociation of H_2_, and the adsorbed hydrogen atoms can be spilled over onto the surface of Mg nanoparticles, which is assigned as a “spillover” mechanism (Zaluskia et al., 1995). In addition, Pd may act as the “nanoportal” role, and hydride nucleation occurs only under Pd nanoparticles at lower hydrogenation temperature (Chung et al., 2015). Hence, it can be concluded that Pd homogeneously distributed on the surface or inside Mg particles can dissociate hydrogen and create a hydrogen diffusion pathway for the Mg nanoparticles.

## Conclusions

In this work, a Mg–Pd nanocomposite was successfully coprecipitated from a homogeneous THF solution. STEM observations revealed that Pd was homogeneously distributed on the surface or inside Mg particles, with the particle size ranging from 40 to 70 nm. The Mg–Pd nanocomposite exhibits superior hydrogen storage properties. The hydrogen reaction enthalpies of the Mg–Pd nanocomposite are slightly lower than that of pristine MgH_2_, indicating that the Mg–Pd alloy plays an active role in the reversible metal hydride transformation and destabilizes the MgH_2_. For the hydrogenated Mg–Pd nanocomposite at 150°C, the onset dehydrogenation temperature is reduced to 216.8°C, representing a 94.3°C reduction as compared with the pure MgH_2_ prepared through the same method. High-content γ-MgH_2_ along with PH_0.706_ is formed during the hydrogenation process, contributing to the enhancing desorption kinetics. The Mg–Pd nanocomposite can take up 3.0 wt% hydrogen in 2 h at temperature low as 50°C. During lower hydrogenation temperatures, Pd can dissociate hydrogen and create a hydrogen diffusion pathway for the Mg nanoparticles.

## Data Availability Statement

All datasets generated for this study are included in the article/supplementary material.

## Author Contributions

The manuscript was written through contributions of all authors. All authors have given approval to the final version of the manuscript.

### Conflict of Interest

The authors declare that the research was conducted in the absence of any commercial or financial relationships that could be construed as a potential conflict of interest.
